# Role of RHEB in Regulating Differentiation Fate of Mesenchymal Stem Cells for Cartilage and Bone Regeneration

**DOI:** 10.3390/ijms18040880

**Published:** 2017-04-24

**Authors:** Sajjad Ashraf, In-Bo Han, Hansoo Park, Soo-Hong Lee

**Affiliations:** 1 Department of Biomedical Science, CHA University, Seongnam-si 463-400, Korea; sajjadashraf471@gmail.com; 2 School of Integrative Engineering, Chung-Ang University, Seoul 156-863, Korea; 3 Department of Neurosurgery, Bundang Medical Center, CHA University, Seongnam-si 463-400, Korea; hanib@cha.ac.kr

**Keywords:** mesenchymal stem cells, Ras homolog enriched in brain (RHEB), differentiation, chondrogenesis, osteogenesis, adipogenesis

## Abstract

Advances in mesenchymal stem cells (MSCs) and cell replacement therapies are promising approaches to treat cartilage and bone defects since substantial differentiation capacities of MSCs match the demands of tissue regeneration. Our understanding of the dynamic process requiring indispensable differentiation of MSCs remains limited. Herein, we describe the role of RHEB (Ras homolog enriched in brain) regulating gene signature for differentiation of human adipose derived mesenchymal stem cells (ASCs) into chondrogenic, osteogenic, and adipogenic lineages. RHEB-overexpression increases the proliferation of the ASCs. RHEB enhances the chondrogenic differentiation of ASCs in 3D culture via upregulation of SOX9 with concomitant increase in glycosaminoglycans (GAGs), and type II collagen (COL2). RHEB increases the osteogenesis via upregulation of runt related transcription factor 2 (RUNX2) with an increase in the calcium and phosphate contents. RHEB also increases the expression of osteogenic markers, osteonectin and osteopontin. RHEB knockdown ASCs were incapable of expressing sufficient SRY (Sex determining region Y)-box 9 (SOX9) and RUNX2, and therefore had decreased chondrogenic and osteogenic differentiation. RHEB-overexpression impaired ASCs differentiation into adipogenic lineage, through downregulation of CCAAT/enhancer binding protein beta (C/EBPβ). Conversely, RHEB knockdown abolished the negative regulation of adipogenesis. We demonstrate that RHEB is a novel regulator, with a critical role in ASCs lineage determination, and RHEB-modulated ASCs may be useful as a cell therapy for cartilage and bone defect treatments.

## 1. Introduction

Multipotent mesenchymal stem cells (MSCs) can potentially differentiate into multiple distinct cell lineages, including chondrogenic, osteogenic, and adipogenic [1]. Since their original discovery in the bone marrow, multiple additional tissue sources, including adipose depots, have been reported to contain MSCs. Adipose tissue in particular is an attractive source of MSCs because it is readily obtainable with minimal morbidity via routine liposuction procedures [2,3]. Adipose-derived stem cells (ASCs), isolated from adipose tissue, are MSCs and thus exhibit multipotency [4]. Additionally, the clinical, tissue engineering, and regenerative applications of in vitro ASC differentiation have been studied extensively [5].

Multiple factors influence the differentiation potential of ASCs into distinct lineages, including the composition of differentiation media, the presence of growth factors, and the substrate properties of the culture plate [6,7]. For instance, ASCs differentiate toward the adipogenic lineage in the presence of dexamethasone (Dex), insulin, 3-isobutyl-1-methylxanthine (IBMX), and indomethacin (Indo), but differentiate towards the osteogenic lineage when a combination of Dex, Glutamax, and β-glycerophosphate (β-GP) is used [8]. The induction of ASCs differentiation into the chondrogenic lineage is usually promoted by a cocktail of Dex, Transforming growth factor (TGF-β1), ascorbic acid, and insulin-transferrin-selenium (ITS) [7,9]. Although the induction of ASC differentiation by numerous factors has been investigated, most studies focus only on differentiation into individual lineages rather than simultaneous chondrogenic, osteogenic, and adipogenic differentiation.

The role of various growth factors in ASC differentiation, multiple transcription factors and genes have also been investigated. Three transcription factors, PPARγ, RUNX2, and SOX9, were clearly shown to drive ASCs to differentiate into either adipocytes, osteoblasts, or chondrocytes, respectively [10]. In addition, MSCs are being considered as vehicles for gene therapy approaches [11]. This knowledge is vital for the development of novel approaches to the inhibition, or enhancement, of expression of either transcription factors or specific lineage markers. The discovery of key transcriptional regulatory factors and their mechanisms could therefore be used to coerce MSCs to differentiate into distinct lineages.

Here, we report a novel role for Ras homolog enriched in brain (RHEB) in the differentiation of ASCs. RHEB is a member of the Ras superfamily of G-Proteins, which regulate cell growth, cell proliferation, and differentiation [12,13]. Previously, we determined the role of RHEB in chondrocytes where RHEB enhances the chondrogenic ability of the chondrocytes via upregulation of SOX9 [13]. However, the role of RHEB in stem cell biology has never been investigated. Therefore, we speculate about whether RHEB also alters the differentiation ability of MSCs towards chondrogenic lineage via SOX9. Additionally, it is well established that during in vivo, cartilage and endochondral bone formation is very closely regulated. Endochondral bone formation first starts with differentiation of MSCs into cartilage and then hypertrophy takes place in cartilage cells that recruit osteoblasts and bone formation occurs [14]. However, adipogenesis has an inverse relationship with the regulation of the osteogensis [10]. Therefore, it is important to understand the role of RHEB in differentiation of ASCs into all three different lineages. Previously, we have reported the multipotency of ASCs via differentiation into distinct lineages [15,16]. During differentiation of ASCs, we found different levels of RHEB mRNA expression in specific lineage determination. Therefore, we examined the mechanisms of RHEB signaling, as well as its role in the differentiation potential of ASCs. These new insights into RHEB function are hugely valuable in the development of gene- and stem cell therapies for regenerative medicine.

## 2. Results

### 2.1. Differential Roles of RHEB Overexpression, Upregulating SOX9 and RUNX2 in ASCs

**Figure 1:**
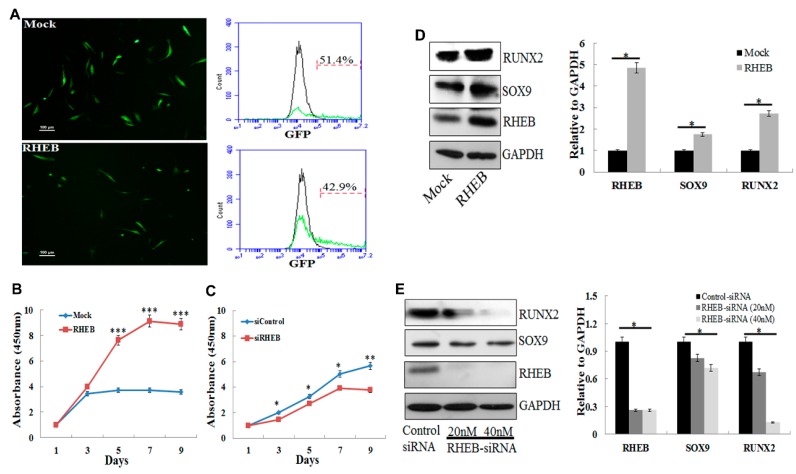
The differential role of Ras homolog enriched in brain (RHEB) in adipose derived stem cells (ASCs). (**A**) **Left panel**: ASCs cells were transfected with Mock and RHEB vectors that harbors sequence for GFP. Transfected cells showed green fluorescent protein (GFP) expression as visualized under fluorescent microscope; **right panel**: Analysis of transfection efficiency by detecting GFP expression thorough flow cytometry; (**B**,**C**) cell proliferation analysis after RHEB overexpression and knockdown, respectively, using CCK-8. During CCK-8 assay, an orange formazan product is produced by cellular dehydrogenases. The amount of formazan produced is directly proportional to the number of living cells. Absorbance of orange formazan was measured at 450 nm; (**D**) **left panel**: protein expression analysis by Western blotting after RHEB overexpression; **right panel**: quantification of the Western blots using ImageJ (1.48v, Wayne Rasband, National Institute of Health, Bethesda, MD, USA) and normalizing with GAPDH; (**E**) **left panel**: protein expression analysis by Western blotting after RHEB knockdown; **right panel**: quantification of the Western blots using ImageJ and normalizing with GAPDH. (Data are shown for a representative experiment as averages of triplicates with standard deviation. The statistical significance of differences was calculated using the Student’s *t*-test. * *p* < 0.05; ** *p* < 0.01; *** *p* < 0.001; Scale bar: 100 μm).

Previously, we described that RHEB imparts a critical role in maintaining chondrogenic characteristics of chondrocytes followed by in vitro and in vivo cartilage formation [12,13]. Here, we investigated whether RHEB plays any role in differentiation of the ASCs into chondrogenic as well as osteogenic cells that are essential for cartilage and bone regeneration. We transfected passage five (P5) ASCs with Mock and RHEB vectors. The mock vector harbors the gene sequence that encodes green fluorescent protein (GFP) and the RHEB vector harbors the gene sequence for RHEB protein as well as for GFP protein. After transfection of the mock vector and RHEB vector, cells expressing mock and RHEB vectors can be visualized by GFP expression under a fluorescent microscope as shown in Figure 1A (left panel). Flow cytometry analysis of the GFP revealed transfection efficiency of 51.4% for the Mock and 42.9% for the RHEB (Figure 1A, right panel). RHEB overexpression significantly increases the proliferation potential of ASCs (Figure 1B) and RHEB silencing using siRNA significantly decreases the proliferation potential of the ASCs (Figure 1C). Hereafter, we evaluated the expression of transcription factors required for chondrogenic and osteogenic differentiation of ASCs in RHEB-overexpressing and RHEB-silenced undifferentiated ASCs cultured in the growth medium. Protein expression confirmed that the RHEB was successfully overexpressed using a microporation technique (Figure 1D). Interestingly, RHEB overexpression also increased the expression of SOX9 and RUNX2 proteins (Figure 1D), these being specific differentiation markers for chondrogenesis and osteogenesis, respectively. The RHEB gene was successfully knocked down by siRNA treatment and RHEB silencing decreased the expression of SOX9 and RUNX2 (Figure 1E). Based on these results, we hypothesize that RHEB has a potential to alter the ability of ASCs to differentiate into distinct lineages.

### 2.2. Effect of RHEB Overexpression and Knockdown on Chondrogenic Differentiation of ASCs

**Figure 2:**
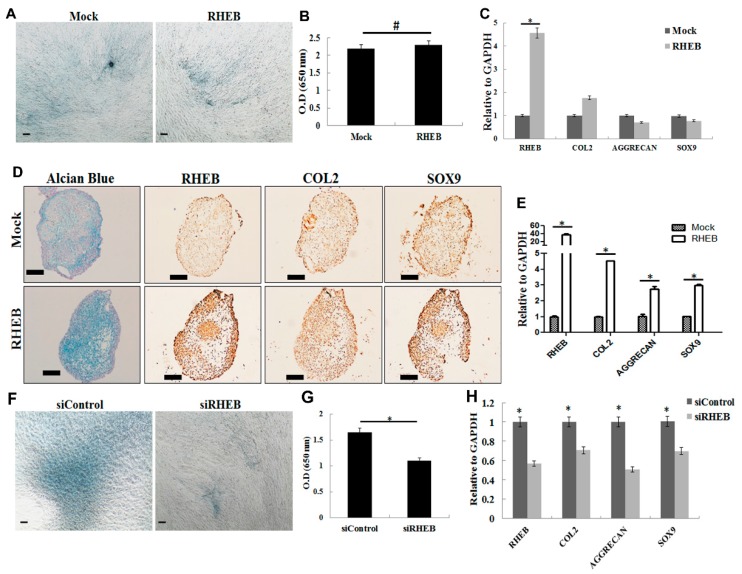
Effect of *RHEB* overexpression and knockdown on chondrogenic differentiation of ASCs in differentiation media. (**A**) Alcian blue staining in monolayer culture of ASCs after *RHEB* overexpression for evaluating glycosaminoglycan (GAG) matrix; (**B**) quantification of the Alcian blue staining extraction from monolayer cultured cells; (**C**) the mRNA analysis by quantitative real-time PCR from monolayer cultured ASCs; (**D**) pellet culture; Alcian blue staining, immunohistochemistry (IHC) was performed for determination of RHEB, COL2 and SOX9 expression; (**E**) the mRNA expression analysis by quantitative real-time PCR in pellet culture; (**F**) Alcian blue staining in monolayer culture ASCs after *RHEB* knockdown; (**G**) quantification of the Alcian blue staining extraction in *RHEB* knockdown ASCs; (**H**) the mRNA expression analysis in monolayer cells after *RHEB* knockdown. (Data are shown for a representative experiment as averages of triplicates with standard deviation. The statistical significance of differences was calculated using the Student’s *t*-test. * *p* < 0.05; Scale bar: 100 μm).

To evaluate the chondrogenic effect of RHEB, P5-ASCs were transfected with mock vector or RHEB harboring vector, and cultured for 21 days in monolayer. In monolayer culture, no marked difference was found in extracellular matrix glycosaminoglycans (GAGs) as indicated by Alcian blue staining (Figure 2A) and quantification of the extracted Alcian blue staining (Figure 2B). Quantitative analysis of mRNA showed overexpression of the *RHEB* gene as well as an increasing trend of *COL2* but no significant change in the expression of *Aggrecan* (*AGG*) and *SOX9* mRNA was found (Figure 2C). There is a controversy about the capacity of the MSCs to differentiate into chondrogenic lineage in vitro monolayer and 3D culture. Therefore, we investigated the effect of RHEB chondrogenic differentiation of ASCs during 3D pellet culture. Surprisingly, the *RHEB*-overexpressing cell pellet showed a more intense blue color after Alcian blue staining, indicating increased GAGs formation (Figure 2D). Additionally, immunohistochemistry analysis revealed increased expression of RHEB, COL2, and SOX9 (Figure 2D). Furthermore, mRNA analysis of chondrogenic markers such as *AGG*, *COL2*, and *SOX9*, as well as *RHEB*, showed higher expression in the *RHEB*-overexpressing cells (Figure 2E). Taken together, these results demonstrate that the upregulation of RHEB increases the chondrogenic differentiation of ASCs in the pellet culture.

The effect of *RHEB* gene expression on the chondrogenic differentiation of ASCs was also investigated by *RHEB* knockdown. A lower level of GAG-matrix formation, indicating a reduction in chondrogenic differentiation potential, was seen in RHEB-depleted ASCs than in controls (Figure 2F). This was confirmed by the quantification of Alcian blue stain extracted from cells (Figure 2G). Quantitative analysis of mRNA expression confirmed significantly lower levels of the chondrogenic markers *COL2*, *AGG*, and *SOX9* following *RHEB* knockdown (Figure 2H). These results validate the RHEB-overexpression results and show the critical role of RHEB in chondrogenesis.

### 2.3. Effect of RHEB Overexpression and Knockdown on Osteogenic Differentiation of ASCs

**Figure 3:**
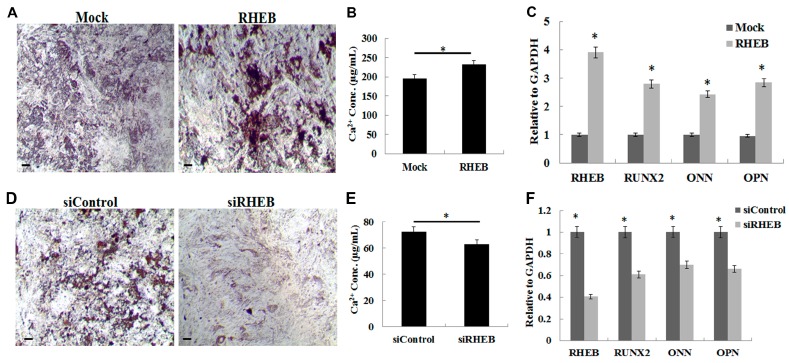
Effect of *RHEB* overexpression and knockdown on osteogenic differentiation of ASCs in differentiation media. (**A**) von Kossa staining showed higher phosphate and calcium mineralization in *RHEB*-overexpressed ASCs; (**B**) estimation of calcium concentration in the cells after *RHEB* overexpression; (**C**) the mRNA expression analysis by quantitative real-time PCR after *RHEB* overexpression; (**D**) von Kossa staining after *RHEB* knockdown in ASCs; (**E**) estimation of calcium concentration in the cells after *RHEB* knockdown; (**F**) the mRNA expression analysis by quantitative real-time PCR after *RHEB* knockdown. (Data are shown for a representative experiment as averages of triplicates with standard deviation. The statistical significance of differences was calculated using the Student’s *t*-test. * *p* < 0.05; Scale bar: 100 μm).

To investigate the involvement of RHEB in commitment to osteogenic differentiation, *RHEB*-transfected ASCs were cultured with osteogenic medium. The higher intensity of von Kossa staining in RHEB-overexpressing ASCs than in controls indicated higher phosphate and calcium contents and depicted greater osteogenic differentiation (Figure 3A). In addition, we also quantified the calcium concentration after osteogenic differentiation of ASCs and found significantly higher calcium levels in *RHEB*-overexpressing ASCs (Figure 3B). Calcium deposition in cells has been observed after 21 days of differentiation, and is thus a late stage indicator of osteogenic differentiation [17]. The increase in such osteogenic differentiation in *RHEB*-overexpressing ASCs was confirmed by analysis of molecular markers. The stable transfection of the *RHEB* gene was confirmed by higher *RHEB* mRNA expression levels (Figure 3C). Additionally, the levels of mRNA expression of the osteogenic markers *osteopontin* (*OP*), *osteonectin* (*ON*), plus the osteogenic transcription factor *RUNX2*, were higher in *RHEB*-overexpressing osteogenic differentiated cells (Figure 3C). Following osteogenic differentiation, the higher expression of osteogenic markers in *RHEB*-overexpressing ASCs correlates well with the higher calcium concentrations seen with von Kossa staining.

In order to validate the increased ASCs osteogenic differentiation seen with *RHEB* overexpression, the induction of osteogenic differentiation of ASCs was performed after *RHEB* silencing, and the level of osteogenesis was examined. A marked reduction in von Kossa staining intensity, indicating reduced osteogenic differentiation and consequently lower calcium deposition, was observed in *RHEB*-knockdown ASCs compared to controls (Figure 3D,E). *RHEB*-silenced osteogenic differentiated cells displayed marked decreases in expression of *RHEB* mRNA, as well as decreased expression of the osteogenic markers *OP*, *ON*, and *RUNX2* (Figure 3F). Overall, data obtained from the overexpression and knockdown of *RHEB* in ASCs indicates that *RHEB* is crucial for osteogenic differentiation.

### 2.4. Effect of RHEB Overexpression and Knockdown on Adipogenic Differentiation of ASCs

**Figure 4:**
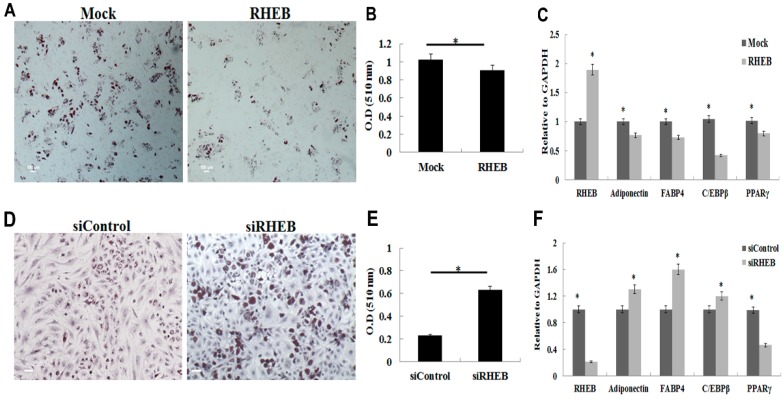
Effect of *RHEB* overexpression and knockdown on adipogenic differentiation of the ASCs. (**A**) Oil Red O staining as seen by higher levels of red colored fat droplets in mock-differentiated cells as compared to *RHEB*-differentiated cells; (**B**) quantification of extracted Oil Red O staining; (**C**) the mRNA expression analysis by quantitative real-time PCR after *RHEB* overexpression; (**D**) Oil Red O staining after *RHEB* knockdown; (**E**) quantification of extracted Oil Red O staining; (**F**) the mRNA expression analysis by quantitative real-time PCR after *RHEB* knockdwon. (Datas are shown for a representative experiment as averages of triplicates with standard deviation. The statistical significance of differences was calculated using the Student’s *t*-test. * *p* < 0.05; Scale bar: 100 μm).

ASCs can differentiate into multiple mesenchymal lineages, including osteogenic, chondrogenic, adipogenic, myogenic, and neurogenic lineages [2,15,16]. Therefore, we evaluated the role of RHEB in adipogenic differentiation of ASCs. *RHEB* overexpression markedly suppressed the ASCs adipogenic differentiation when adipogenic differentiating medium was used. Oil Red O staining was performed to evaluate adipose depots, and *RHEB*-overexpressing cells were found to have lower levels of positive staining than mock-transfected cells (Figure 4A). Quantification of the Oil Red O stain extracted from cells also revealed significantly less staining in the *RHEB*-overexpressing differentiated ASCs than in the controls (Figure 4B). Additionally, *RHEB* overexpression was associated with lower mRNA expression of the adipogenesis-related genes *Adiponectin*, *FABP4*, *PPARγ*, and *C/EBPβ* (Figure 4C). These results indicate that RHEB plays a crucial role in impairing the adipogenic differentiation of ASCs.

The adipogenic differentiation capacity of ASCs following *RHEB* knockdown was investigated. siRNA knockdown of RHEB in ASCs was associated with higher intensity of Oil Red O staining, indicating increased adipogenic differentiation (Figure 4D). Quantification of Oil Red O stained extracts revealed an approximately three-fold higher intensity in the adipogenically differentiated cells in which *RHEB* was silenced (Figure 4E). Following 14 days of adipogenic differentiation and *RHEB* knockdown, the expression of *RHEB* mRNA was markedly reduced, whereas that of adipogenic markers such as *adiponectin*, *FABP4*, and *C/EBPβ* was increased (Figure 4F). Interestingly, expression of PPARγ, an adipogenic marker, was decreased (Figure 4F). These results indicate that RHEB can potentially regulate the adipogenic differentiation of ASCs in differentiating medium. Overall results indicate that RHEB plays a crucial role in identification and differentiation of ASCs into distinct lineages.

## 3. Discussion

Human ASCs have the potential to differentiate into specific lineages. The induction of ASC differentiation towards a specific mesenchymal lineage is dependent on changes in gene expression, which gradually alter the ASC phenotype until it is characteristic of the target cell [2]. Various theories about ASC differentiation have been proposed, and, consequently, numerous molecular markers have been evaluated [18]. The mechanisms controlling chondrogenic, osteogenic, and adipogenic differentiation of ASCs are among the most important and physiologically relevant differentiation processes, and the proper control of differentiation into these three lineages is vitally important for regulation of cartilage, bone, and fat tissue regeneration [19].

The differentiation of ASCs has mainly been studied by culturing cells in specific differentiation medium, supplemented with a classical cocktail of induction factors, to eventually induce the in vitro differentiation into chondrogenic, osteogenic, or adipogenic lineages. However, the differentiation capacity of ASCs is not always reproducible. In this study, we have evaluated the role of the cell cycle- and growth-associated gene RHEB in the differentiation of ASCs into three individual distinct lineages. For comparison, we also investigated the effects of RHEB on ASCs in nondifferentiating medium. We discovered that RHEB regulates genes associated with chondrogenesis and osteogenesis in nondifferentiating medium (Figure 1). We thus hypothesized that RHEB is a regulator of differentiation.

To confirm the role of RHEB in ASC differentiation, ASCs were cultured in various differentiation media following the overexpression or knockdown of RHEB. We have shown that RHEB plays a critical role in the regulation of ASC differentiation, wherein, interestingly, the over-expression of RHEB decreased adipogenesis but increased chondrogenesis and osteogenesis from ASCs precursors. As expected, the opposite effects were observed on RHEB-knockdown.

Interestingly, RHEB overexpression clearly increased chondrogenesis using the pellet culture format (Figure 2), but no such effect was seen when a monolayer culture was used. It is well established that the pellet culture format provides more of the cell–cell interactions that are needed for the terminal differentiation of ASCs into the chondrogenic lineage [20,21]. Chondrogenic medium contains TGF-β1 to enhance chondrogenesis, but our study shows that RHEB-overexpression markedly increases chondrogenic differentiation. In our case, RHEB most likely induces chondrogenesis through the regulation of SOX9, a key transcription factor for COL2 that is involved in aggrecan synthesis [22,23,24]. Conversely, the effect of *RHEB* gene silencing on chondrogenesis was only evaluated after monolayer culture but not by 3D culture because of certain experimental limitations. The *RHEB* gene was properly silenced throughout the differentiation period of 21 days with siRNA treatment on every third day. It is well documented that siRNA effects last for 5–7 days. Therefore, to achieve the proper effect of *RHEB* silencing, siRNA treatment at multiple times throughout the experiment was required. However, siRNA cannot penetrate deep into the pellet, and, thus, would possibly give the wrong conclusion. Interestingly, RHEB silencing downregulates the chondrogenesis even in 2D culture (Figure 2F) and obviates the need of 3D culture.

On the other hand, the increase in osteogenic differentiation by the *RHEB* gene can be attributed to its regulation of RUNX2. The expression of RHEB and RUNX2, a key regulator of osteogenesis, are highly correlated [25]. Mineralization, resulting in the accumulation of calcium and phosphate ions, is a feature of late stage osteogenesis. We observed higher levels of mineralization with overexpression of RHEB, shown by von Kossa staining and quantitative calcium analysis, indicating that RHEB also plays a role in late stage osteogenesis.

Unlike with osteogenesis and chondrogenesis, *RHEB*-overexpression decreased the adipogenic differentiation of ASCs, with the converse seen in *RHEB*-knockdown. This negative effect on adipogenesis was probably associated with C/EBPβ and PPAR-γ expression. We found that *RHEB*-knockdown increased the expression of C/EBPβ in ASCs cultured in nondifferentiating medium (data not shown). Several studies have reported a strong inverse correlation between osteogenesis and adipogenesis [26,27]. Previously, Hong et al. reported a transcriptional modulator of mesenchymal stem cells, known as transcriptional co-activator with PDZ-binding motif (TAZ), that can simultaneously regulate osteogenesis and adipogenesis via the upregulation of RUNX2 and the repression of PPARγ [10], which is consistent with our results on the effects of *RHEB* gene regulation on ASCs differentiation. However, during Adipogenesis after RHEB knockdown, expression of C/EBPβ increases while PPAR-γ decreases. It has been previously reported that C/EBPβ is responsible for upregulation of the PPAR-γ at terminal stages of Adipogenesis [28]. On the contrary, in our studies, we found the opposite effect on C/EBPβ and PPAR-γ after RHEB knockdown. The possible reason for these results would be the regulation of PPAR-γ by mammalian target of rapamycin (mTOR), as mTOR is a downstream target of the RHEB [29] and also responsible for the upregulation of the PPAR-γ. After *RHEB* knockdown, it might be mTOR level decreases that ultimately downregulated PPAR-γ [30]. However, C/EBPβ expression is independent of the mTOR pathway as shown by Kim and Chen [30] via reporter assay. Although previous studies supported our findings, further experiments are still needed to find out the regulatory mechanism in depth. There are also some other limitations in this study, as we only analyzed the chondrogenesis, osteogenesis, and adipogenesis at endpoint in each case. Analysis across a time-course of differentiation would be valuable for identifying the mechanism of RHEB. We also observed a small difference in mRNA expression of certain genes as shown in Figure 3 and Figure 4, but that difference was statistically significant with a *p*-value < 0.05. This small difference might be because of analysis of three patients’ samples. In order to achieve high significant differences of gene expressions among control and treated groups, samples from more patients need to be analyzed.

A schematic approach of RHEB role in ASCs is shown in graphical abstract. Further studies are required in order to elucidate the complex mechanisms underlying the role of RHEB in differentiation, including whether RHEB acts directly as a transcriptional factor or through the modulation of other genes. However, RHEB enhancing chondrogenic and osteogenic differentiation of the stem cells is a good indication to use modulated stem cells for treatment of cartilage and bone defects.

## 4. Materials and Methods

### 4.1. Adipose-Derived Stem Cell Culture

Adipose tissue was collected via liposuction from the knees of three patients, with approval of the ethics committee of CHA University (13 May 2015) and institutional review board (IRB) issued IRB No. BD2014-096. Written informed consent was obtained from all patients. All three patients were females above 50 years of age. ASCs were harvested from adipose tissue isolated from different patients separately. Isolated adipose tissue was washed thrice with phosphate-buffered saline (PBS). After washing, tissue was digested with 0.5 mg/mL collagenase (Sigma Aldrich, St. Louis, MO, USA) diluted in Dulbecco’s Modified Eagle Medium (DMEM) low-glucose media (Hyclone, GE Healthcare Life Sciences, South Logan, UT, USA) containing 10% fetal bovine serum (FBS) and 1% penicillin/streptomycin (P/S), for 45 min, with frequent shaking at 37 °C. Digested tissue was then dissected with sterile scissors. Cleaved tissue was centrifuged at 1000 rpm for 5 min, and the fatty supernatant was removed. The remaining solution was filtered through a 40-μm strainer and then centrifuged at 1000 rpm for 5 min. After centrifugation, isolated ASCs were resuspended in 15 mL of DMEM low-glucose medium (DMEM-LG) supplemented with 10% FBS and 1% P/S, and cultured in 150 mm culture dish (Corning incorporated, Corning, NY, USA) in monolayer at 37 °C in a 5% CO_2_ incubator. The growth medium was replaced every third day. Isolated ASCs were either cultured for expansion until passage 5 (P5) or cryopreserved in liquid nitrogen for further use. For monolayer culture, ASCs were cultured in DMEM-LG supplemented with 10% (*v*/*v*) fetal bovine serum (FBS) and 100 units/mL of P/S in humidified air with 5% (*v*/*v*) CO_2_ at 37 °C and with an initial seeding density of 2 × 10^4^ cells/cm^2^ until unless seeding density mention for specific experiments.

### 4.2. Gene Delivery

A human RHEB (accession number: NM_005614.3) expression vector was purchased from Enzynomics (Daejeon, Korea). Briefly, the human *RHEB* gene was cloned in the pEGFP-N1 vector, which also contained the green fluorescent protein (GFP) gene. As a control, pEGFP-N1 was used as a Mock vector without the *RHEB* gene sequence but with GFP. A NeonTM Microporator (Invitrogen, Carlsbad, CA, USA) was used for transfection of the RHEB gene into ASCs as follows. Briefly, 1 × 10^6^ P5 ASCs were transfected with 5 μg of plasmid DNA, using 100 μL of “R” buffer. Microporator conditions were optimized for transfection, and used as follows: 1150 V for 30 ms with two pulses, for either the mock transfection (control), or transfection of the RHEB vector. Transfection efficiency was evaluated 24 h after transfection by measuring GFP expression, using an Eclipse 55i microscope (Nikon, Kanagawa, Japan). GFP expression was also quantified by flow cytometry with a FACSCalibur system (BD, San Jose, CA, USA). Briefly, transfected cells were trypsinized and washed with PBS supplemented with 1% FBS. After washing, cells were resuspended in PBS and analyzed by flow cytometer.

### 4.3. RHEB Knockdown

RHEB knockdown was achieved using duplex small interfering RNA (siRNA) (Origene Technologies, Rockville, MD, USA). P5 ASCs were transfected with either 20 nmol or 40 nmol RHEB siRNA, or control siRNA, using Lipofectamine RNAi Max reagent (Invitrogen) according to the manufacturer’s instructions. Following siRNA treatment, RHEB expression in ASCs was evaluated using PCR and Western blotting, after 48 or 72 h of incubation, respectively. For silencing of the RHEB gene throughout differentiation of the ASCs into adipogenic, chondrogenic, and osteogenic lineage, we treated the cells with siRNA on every third day before changing the specific differentiation media.

### 4.4. Adipogenic Differentiation of ASCs and Oil Red O Staining

P5 ASCs were cultured as a monolayer, with an initial seeding density of 2 × 10^4^ cells/cm^2^. After 24 h, typical growth medium was replaced with adipogenic differentiation medium, composed of DMEM high-glucose medium containing FBS (10%), insulin (10 μg/mL), IBMX (500 μM), indomethacin (200 μM), Dex (1 μM) and P/S (1%). Adipogenic medium was refreshed every third day for 14 days.

Following 14 days of adipogenic differentiation, medium was removed, and cells were washed three times with PBS before being fixed in 10% formalin. Cells were then washed once with PBS and three times with 60% isopropanol. Cells were incubated with Oil Red O stain (0.5% in isopropanol) for 30 min before being washed three times with 60% isopropanol and once with distilled water (DW). Hematoxylin was then added, and the cells were stained for 30–60 s before washing them with tap water. DW was then added, and images were taken.

Oil Red O stain was extracted from cells by soaking in 100% isopropanol for 15 min. Two hundred microliters of extracted stain was then aspirated and added to a 96-well ELISA plate before reading at 510 nm.

### 4.5. Chondrogenic Differentiation of Adipose Stem Cells

P5 ASCs were cultured in a monolayer up to 95% confluency, before being trypsinized, suspended in DMEM low-glucose medium, and pelleted by centrifugation at 1200 rpm for 3 min. Supernatant was aspirated, and the cells were resuspended in fresh DMEM low-glucose medium. For pellet culture, 2 × 10^5^ cells were centrifuged at 1500 rpm for 1 min in a 15 mL conical tube (SPL Life Sciences, Gyung-gi, Korea), and then incubated at 37 °C for 21 days in humidified air with 5% (*v*/*v*) CO_2_.

Chondrogenic differentiation was induced by incubating the cell pellets in DMEM high-glucose medium supplemented with 10% FBS, 0.5% ITS, 50 µg/mL ascorbic acid, 100 nm Dex, 0.5% P/S, and 10 ng/mL TGF-β1. The chondrogenic medium was refreshed every two days for 21 days.

### 4.6. Alcian Blue Staining and Immunohistochemistry

In vitro pellet culture samples, obtained after 21 days, were fixed in 4% (*w*/*v*) paraformaldehyde (PFA; Biosesang, Seongnam-si, Korea). After processing, specimens were embedded in paraffin wax, sliced into 4-μm thick sections, and stained with 1% Alcian blue stain in 3% acetic acid (pH 2.5) for 30 min, before counter-staining with nuclear fast red for 5 min. The glycosaminoglycan (GAG) matrix, formed in pellet culture, was observed using light microscopy.

For immunohistochemistry analysis, tissue sections were deparaffinized and hydrated using sequential xylene and ethanol incubations, before being treated with 3% H_2_O_2_ in methanol for 10 min. Samples were then washed, treated with pepsin (1 mg/mL in 10 mmol HCl) at 37 °C for 10 min, and blocked with 10% goat serum for 1 h. Slides were then washed with PBS, and incubated with monoclonal primary antibody for RHEB (Abcam, ab25873), Sox9 (Abcam, ab3697), and COL2 (Millipore, CP-18, Darmstadt, Germany), for 18 h at 4 °C, in a humidified chamber. Following overnight incubation, slides were washed with PBST before being incubated with biotinylated secondary antibody for 30 min, followed by streptavidin-HRP for 30 min. Color was developed by treating with diaminobenzidine (DAB) for 5–10 min. Hematoxylin (Sigma Aldrich) was used as a counter-stain. Stained samples were mounted in mounting medium and visualized using a light microscope.

### 4.7. Osteogenic Differentiation of ASCs and Von Kossa Staining

P5 ASCs were cultured in a monolayer, with an initial seeding density of 2 × 10^4^ cells/cm^2^. After 24 h, growth medium was replaced with osteogenic differentiation medium, composed of DMEM high-glucose medium containing 10% FBS, 10 mM glycerol-2-phosphate, 50 μg/mL ascorbic acid, 100 nM Dex, 1X Glutamax and 1% P/S. Osteogenic medium was refreshed every three days for 21 days.

For von Kossa staining, after 21 days of osteogenic differentiation, the medium was removed and cells were washed three times with PBS before fixing with 10% formalin. Once fixed, the cells were washed once with PBS and three times with DW, before being incubated with 5% silver nitrate solution for 1 h under strong light. Cells were then washed three times with DW before being treated with nuclear fast red (NFR) for 5 min. DW was then added to wells and images were taken.

### 4.8. Calcium Concentration Estimation

The calcium concentration in cells undergoing osteogenic differentiation was estimated using the QuantiChrom^TM^ Calcium Assay Kit (Cat. No. DICA-500, BioAssay Systems, Hayward, CA, USA). Briefly, after 21 days of differentiation in a 24-well plate, the cells were washed with PBS and then fixed with 10% formalin for 20 min. Cells were then incubated in PBS for 15–20 min at 4 °C. PBS was removed, and cells were stored at −80 °C for 20 min before being freeze-dried for 30 min at −50 °C. The cells in each well were subsequently soaked in 200 µL of working reagent, for 3 min at room temperature. The optical density was measured at 570–650 nm, and the concentration was determined using a standard curve.

### 4.9. PCR

Total RNA was extracted using TRIzol reagent (Invitrogen), and cDNA was synthesized using an AccuPower RT-PreMix kit (Bioneer, Daejeon, Korea). Real-time quantitative PCR (qPCR) was performed using Power SYBR Green PCR master mix (Life Technologies, Carlsbad, CA, USA) with the StepOnePlus real-time PCR system (Applied Biosystems, Waltham, MA, USA). Expression of all genes was normalized to the housekeeping gene, GAPDH. Primer details are shown in Table S1.

### 4.10. Western Blotting

Protein was isolated from cultured cells using a radioimmunoprecipitation assay (RIPA) buffer (Sigma Aldrich), and its concentration subsequently measured using the bicinchoninic acid assay (Pierce, Rockford, IL, USA), according to the manufacturer’s instructions. Approximately 25 μg of total protein was separated by sodium dodecyl sulfate-polyacrylamide gel electrophoresis (SDS-PAGE), and western blotting was performed using standard procedures, as described previously [31]. Quantification was performed using ImageJ software as described previously [32]. Briefly, measurements of the protein bands were normalized with the measurement of the GAPDH and data was plotted. Anti-RHEB, anti-SOX9 and anti-GAPDH antibodies were purchased from Abcam (Cambridge, MA, USA), and anti-RUNX2 antibody was purchased from Santa Cruz Biotechnology, Inc. (Dallas, TX, USA). Alexa Fluor 594-conjugated goat anti-mouse and anti-rabbit IgG antibodies were purchased from Molecular Probes (Eugene, OR, USA).

### 4.11. Cell Proliferation Assay

A Cell Counting Kit-8 assay kit (Dojindo Laboratories, Kumamoto, Japan) was used to perform cell proliferation assays. Briefly, P5 transfected ASCs were seeded at a density of 1 × 10^4^/cm^2^, and incubated for 7 days in a 24-well plate. Cell proliferation was measured on alternate days, as per the kit’s standard protocol.

### 4.12. Statistical Analysis

All experiments were performed with the approval of the CHA University Ethics Committee (IRB NO. PBC09-099). Experiments using each condition were performed on three biological samples and in triplicate, and results are expressed as mean ± standard deviation. One-way analysis of variance (ANOVA) was used for analysis of quantitative values, and Tukey’s post hoc test was used for all pairwise comparisons between groups. In addition, *p*-values < 0.05 were considered statistically significant.

## 5. Conclusions

We have demonstrated that RHEB plays a crucial role in the differentiation of ASCs, wherein it promotes chondrogenic and osteogenic differentiation but impairs adipogenesis. Our findings indicate that RHEB regulated chondrogenesis and osteogenesis critically controls SOX9 and RUNX2 expression, respectively. Conversely, a decrease in ASCs adipogenesis was due to the downregulation of C/EBPβ. It is likely that RHEB acts as a molecular switch between RUNX2 and C/EBPβ, in order to fine-tune the balance between osteogenesis and adipogenesis. Therefore, we conclude that the modulation of RHEB expression in ASCs could prove useful in stem cell therapies for the regeneration of tissues such as cartilage and bone.

## Supplementary Materials

Supplementary materials can be found at www.mdpi.com/1422-0067/18/4/880/s1.
