# Supplementary material for: Role of RHEB in Regulating Differentiation Fate of Mesenchymal Stem Cells for Cartilage and Bone Regeneration
Source: Int J Mol Sci. 2017 Apr 24;18(4):880. doi: 10.3390/ijms18040880 (PMC5412461; doi:10.3390/ijms18040880)
Supplement: Supplementary file 1 [file ijms-18-00880-s001.pdf]

Table S1. Detail of primers for quantitative real time PCR (qPCR).

Gene Names	Primer Sequence	Product Size (bp)
GAPDH (NM_002046)	F: 5′-ACATCGCTCAGACACCATG-3′<br>R: 5′-TGTAGTTGAGGTCAATGAAGGG-3′	143
RHEB (NM_005614.3)	F: 5′-GAGTCCACAAATTGGCCTTC-3′<br>R: 5′-CAGTCCAAGTCCCGGAAGAT-3′	95
COL2 (NM_001844)	F: 5′-CACGTACACTGCCCTGAAGGA-3′<br>R: 5′-CGATAACAGTCTTGCCCCACTT-3′	65
SOX9 (NM_000346)	F: 5′-GTACCCGCACTTGCACAAC-3′<br>R: 5′-TCTCGCTCTCGTTCAGAAGTC-3′	74
Aggrecan (NM_013227)	F: 5′-GCCTGCGCTCCAATGACT-3′<br>R: 5′-ATGGAACACGATGCCTTTCAC-3′	104
Adiponectin (NM_004797)	F: 5′-TTCCATACCAGAGGGGCTCA-3′<br>R: 5′-CCCTTGAGTCGTGGTTTCCT-3′	94
FABP4 (NM_001442.2)	F: 5′-GCATGGCCAAACCTAACATGA-3′<br>R: 5′-CCTGGCCCAGTATGAAGGAAA-3′	107
CEBPβ (NM_005194.2)	F: 5′-*GCAAGAGCCGCGACAAG*-3′<br>R: 5′-*GGCTCGGGCAGCTGCTT*-3′	154
PPARγ (NM_015869.4)	F: 5′-*GATACACTGTCTGCAAACATATCACAA*-3′<br>R: 5′-CCACGGAGCTGATCCCAA-3′	91
RUNX2 (NM_004348)	F: 5′-CAGACCAGCAGCACTCCATA-3′<br>R: 5′-CAGCGTCAACACCATCATTC-3′	178
Osteonectin (NM_003118.3)	F: 5′-GAAAGAAGATCCAGGCCCTC-3′<br>R: 5′-CTTCAGACTGCCCGGAGA-3′	90
Osteopontin (NM_001040060)	F: 5′-TTGCAGCCTTCTCAGCCAAA-3′<br>R: 5′-AACTTCGGTTGCTGGCAGGT-3′	459

* F= Forward Primer, * R= Reverse Primer.
